# In-Hospital Mortality and Its Predictors among Hospitalized Diabetes Patients: A Prospective Observational Study

**DOI:** 10.1155/2023/9367483

**Published:** 2023-03-30

**Authors:** Dereje Eyob Tediso, Fekede Bekele Daba, Teshale Ayele Mega

**Affiliations:** ^1^Jimma University, College of Health Sciences, School of Pharmacy, Jimma, Ethiopia; ^2^Addis Ababa University, College of Health Sciences, School of Pharmacy, Department of Pharmacology and Clinical Pharmacy, Addis Ababa, Ethiopia

## Abstract

**Background:**

Diabetes mellitus (DM) is one of the leading health emergencies of the 21^st^ century and among the top ten causes of death among adults globally in 2017. Although Ethiopia has been victimized by the growing prevalence of DM, data regarding in-hospital mortality among admitted diabetic patients in Ethiopia, specifically in Jimma Medical Center (JMC), are lacking.

**Objective:**

The aim of the study is to assess in-hospital mortality and its predictors among DM patients admitted to Jimma Medical Center.

**Methods:**

A hospital-based prospective observational study was employed involving 120 diabetes patients admitted to JMC from October 01, 2020, to June 30, 2021. Data were collected on variables related to the patient, disease, medication, and clinical outcomes. Data were entered into Epidata version 4.6.0.4 for cleaning and exported to SPSS version 23.0 for analysis. Kaplan–Mayer and cox-regression analyses were used to compare the survival experience and to determine the predictors of clinical outcomes, respectively. Hazard ratio with its two-sided *p* value <0.05 was considered to declare the statistical significance.

**Result:**

Of 120 DM patients, 81 (67.5%) of them were males. The in-hospital mortality was 13.34% (16/120). Rural residence (AHR: 3.46; 95% CI (1.12, 9.81)), age (AHR: 1.03; 95% CI: (1.001, 1.059)), admission with diabetic ketoacidosis (AHR: 5.01; 95% CI (1.12, 21.88)), and multiple comorbidities: five comorbidities (AHR: 9.65; 95% CI (1.07, 19.59)) and six comorbidities (AHR: 14.02; 95% CI (1.74, 21.05)) were independently associated with in-hospital mortality. On the other hand, exposure to nonantidiabetic medications decreased the hazard of mortality by 86.5% (AHR: 0.135; 95% CI (0.04, 0.457)).

**Conclusion:**

This study showed the rate of in-hospital mortality was noticeably high. The study showed that rural residence, age, DKA, and having comorbidities (five and six) were the statistically significant predictors of in-hospital mortality. In contrast, the use of nonantidiabetic medications such as statins, ASA, and other antihypertensive agents before admission remained protective. Thus, proper strategies have to be devised to improve in-hospital mortality among admitted DM patients.

## 1. Introduction

Diabetes mellitus is one of the most common medical conditions prevalent all over the globe. In 2021, it is estimated that 537 million people, representing 10.5% of the global adult population, have DM. This number is expected to increase to 643 million (11.3%) in 2030 and 783 million in 2045. African region, including Ethiopia, is anticipated to have the greatest increase in the number of people with diabetes. Diabetes prevalence in Ethiopia in the last 17 years (2000–2016) ranges from 0.3% at Debre Berhan Referral Hospital to 7% in Harar town [1, 2].

Diabetes mellitus is the leading cause of global morbidity and mortality. It attributed for 12.2% of all global deaths between the age of 20–79 years and 32.6% of all deaths occurring in the productive age group. African region, especially Sub-Saharan Africa (SSA), is largely inflicted [3, 4]. International Diabetes Federation reported an estimated 416,163 diabetes-related deaths in Africa, and the majority of those deaths occur in people aged ≤60 years [5]. A study from central Ghana showed in-patient diabetes mortality rates increased from 7.6 per 1000 to 30 per 1000 deaths from 1983 to 2012, respectively. The reported average 28-day mortality rate was 18.5% [6]. In Ethiopia, DM-related mortality rate ranges from 2% to 21% [7, 8]. It is estimated that 26,448 diabetes-related deaths occur in adults 20–79 years, in 2021 [5].

Several studies showed that in-hospital mortality among DM patients was significantly associated with older age, gender, hypertension, hyperlipidemia, burden of comorbidities, infection, poor glycemic control, lack of foot care, long standing diabetes, and prolonged hospital stay [9–11]. Despite different initiatives undertaken by the Ethiopian Diabetes Association and the country's National Strategic Action Plan (NSAP) for prevention and control of noncommunicable disease (NCD) including diabetes, currently, the country has been challenged by the growing magnitude of diabetes. Ethiopia is among the top 4 countries with the highest adult diabetic populations in Sub-Saharan Africa (SSA) [12], but important data regarding in-hospital mortality were inadequate [13]. Therefore, this study was aimed to assess in-hospital mortality and its predictors among patients with DM admitted to JMC.

## 2. Methods and Participants

### 2.1. Study Area and Period

The study was conducted at Jimma Medical Center (JMC). It is located in Jimma town, 352 km southwest of Addis Ababa, the capital city of Ethiopia. It is the only teaching hospital in southwest Ethiopia, providing services for approximately 15,000 inpatients. Moreover, 160,000 outpatients, 11,000 emergency cases, and 4500 deliveries will attend the center annually, and its catchment population is estimated to be over 20 million. This study was conducted from October 01, 2020, to June 30, 2021, at the emergency department, medical, surgical, and gynecology/obstetrics wards of JMC.

### 2.2. Study Design and Population

A hospital-based prospective observational study was employed. All patients admitted to JMC with the diagnosis of DM were considered as source population and admissions to the emergency department, medical, surgical, and gynecology/obstetrics wards with the diagnosis of DM during the data collection period and fulfill the eligibility criteria were considered as sample population.

### 2.3. Eligibility Criteria

Patients aged 18 years and those who spent at least ≥24 hours in the hospital were included in the study. Whereas, patients diagnosed with gestational DM, who refused to participate, and patients or caregivers who were unable to provide appropriate information during data collection were not considered eligible.

### 2.4. Sample Size Determination and Sampling Technique

The sample size for the study was determined by using the single population proportion formula. Considering the proportion of in-hospital mortality rate (*p*=11.2%) by Kefale et al. [14], *Z* (standardized normal distribution value at 95% CI) of 1.96, margin of error (d) of 5%, and 10% nonresponse rate, the initial sample size calculated was 153 patients. However, the number of diabetes patients admitted to the emergency room, medical, surgical, and gynecology/obstetrics wards over nine months in 2019 was only 382. Hence, the finite population correction formula was applied and the corrected sample size became 109 patients. Adding 10% for the nonresponse rate, the final sample size became 120 patients. As the number of admissions was limited during the time of data collection, all admissions who met the inclusion criteria were recruited in the study using a consecutive sampling technique, and every patient was followed until discharge, referral to other facilities, or death.

### 2.5. Study Variables

In-hospital mortality was considered as an outcome variable. Predictor variables include patient-related factors (sociodemographic variables (age, sex, marital status, educational status, residence, and occupation)), disease-related factors (type of DM, duration of DM, length of previous hospital stay, DM-related complications, comorbidities, previous hospitalization, admission blood glucose level, systolic blood pressure (SBP), and diastolic blood pressure (DBP)), and medication-related factors (types of medications (antidiabetics and nonantidiabetic medications) and duration of treatment).

### 2.6. Data Collection Procedures and Quality Control

Once the data collection tool was developed, it was reviewed by a group of endocrinologists and validated based on their recommendation. It was initially designed in English, then translated to the local language (Afan Oromo and Amharic), and back-translated into English by language experts to assure its consistency. The semistructured questionnaire was designed to extract information through face-to-face interviews (sociodemographic data and some parts of the clinical characteristics of the patients), and the patients' medical charts were also reviewed (to extract data on clinical characteristics of the patients uncovered by interviews, clinical outcome, medication prescribed after admission, vital signs, and laboratory data). Data were collected by two trained pharmacists (B. Pharm) and one BSC Nurse; while one medical doctor was assigned to supervise the data collection process. All the protocols of COVID-19 were considered during data collection.

To ensure the data quality, data collectors and a supervisor were trained for one day before starting data collection on: how to collect the data, the contents of the questionnaire, ethical issues, how to obtain additional information from the treating physicians, and patient interviews. The data collectors were also strictly supervised, and the principal investigator reviewed all filled formats daily. Moreover, a pretest was conducted on 5% of the participants before the actual data collection to check the consistency and validity of the data collection tool.

### 2.7. Data Processing and Analysis

Data were entered into Epidata version 4.6.0.4 and exported to Statistical Package for Social Sciences (SPSS) version 23.0 for cleaning and analysis, respectively. Mean and standard deviation (SD) were used to summarize continuous variables. Categorical variables were expressed in percentage and frequency. Descriptive analysis was performed, and the results were presented by the texts, tables, and figures. Kaplan–Meier (log-rank test) was used to compare the survival experience of the patients. The Cox-proportional hazard model was used to determine predictors of in-hospital mortality. Bivariate Cox regression analysis was conducted, and the variables with *p* value less than 0.25 were considered for the multivariable regression analysis. The hazard ratio was used as a measure of the strength of association, and the variables with *p* value <0.05 on the multivariable Cox regression were used to declare statistical significance.

## 3. Results

### 3.1. Overview of the Study Participants

**Figure 1:**
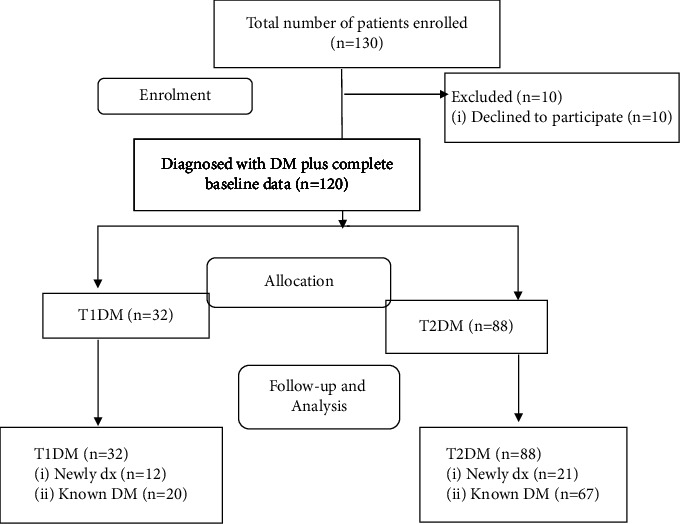
Study flow diagram among patients with DM admitted to JMC from October 01, 2020, to June 30, 2021.

Out of 130 consecutive patients admitted over nine months, 10 patients were excluded and 120 patients were included in the final analysis. Among those included in the analysis, 89 (74.17%) of them were admitted to the medical ward (Figure 1).

### 3.2. Sociodemographic Characteristics

Eighty one (67.5%) of study participants were males. The mean (+SD) age of the participants was 50.21 ± 19.35 years. About one-third (37.5%) of them were farmers, and nearly half 58 (48.3%) of them had no formal education (Table 1).

### 3.3. Reasons for Hospitalization

Diabetic ketoacidosis (DKA) was the most common reason for hospitalization. It accounted for 59 (49.2%) of the admissions. Admissions related to infections were 34 (28.33%) and that of diseases of circulatory system were 16 (13.34%). The most common infection responsible for admission was pneumonia 15 (12.5%), and the most common cardiovascular disease attributed for admission was heart failure 5 (4.17%) (Table 2).

### 3.4. Baseline Blood Glucose Level during Admission

**Figure 2:**
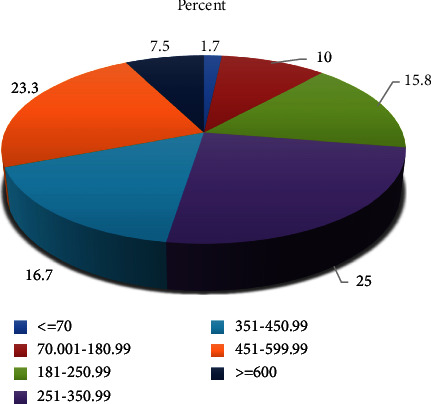
Admission blood glucose (RBS) level among patients with DM admitted to JMC from October 01, 2020, to June 30, 2021.

Of the 120 patients, 87 (72.5%) of them had random blood sugar (RBS) ≥ 251 mg/dl and only two (1.7%) had RBS ≤70 mg/dl during hospital admission (Figure 2).

### 3.5. Disease-Related Factors

**Figure 3:**
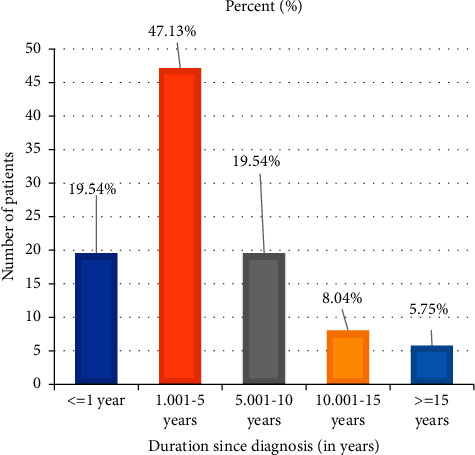
Duration of known diabetes among patients with DM admitted to JMC from October 01, 2020, to June 30, 2021.

From 120 patients, 88 (73.3%) were diagnosed with type 2 DM and 87 (72.5%) patients were known diabetics (Figure 1). The mean duration of diabetes for known diabetics was 3.77 ± 4.67 years. Fifty eight (66.67%) of the known diabetics were diagnosed in the last 5 years (Figure 3), and 80 (91.95%) of them had a regular follow-up. Fifteen (17.24%) patients were attending their follow-up monthly, 42 (48.27%) patients every 2 months, and 23 (26.44%) patients every 3 months. Forty-three (49.43%) patients had at least one prior history of admission to JMC. Among these, 30 (69.77%), 12 (27.91%), and 1 (2.32%) patient had 1, 2, and 3 times a prior history of admission, respectively.

Overall, 87 (72.5%) patients had at least one acute or chronic comorbidity. Hypertension was the most common type of comorbidity contributing to 51 (58.62%) of the cases, followed by pneumonia 35 (40.23%) (Table 3).

**Figure 4:**
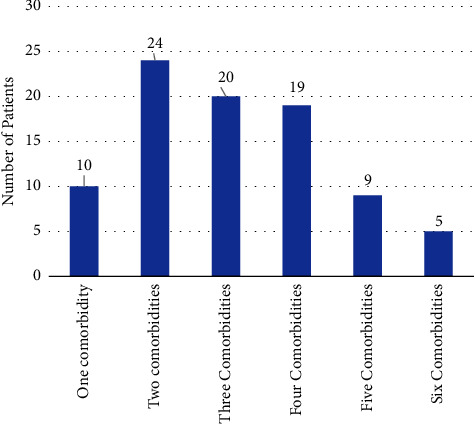
Number of comorbidities among patients with DM admitted to JMC from October 01, 2020, to June 30, 2021.

Among the study participants, 10 (11.49%) patients had one comorbidity, 24 (27.59%) patients had two comorbidities, and 53 (60.92%) patients had >3 comorbidities (Figure 4).

In this study, 67 (55.8%) patients had at least one long-term diabetic complication, and of which42 (62.68%) patients were diagnosed with neuropathy (Table 4).

### 3.6. Medication-Related Factors

Except the 33 newly diagnosed patients, 50 (57.47%) of the study participants were receiving oral glucose-lowering agents, 34 (39.08%) were on neutral protamine hagedorn (NPH) insulin, and 50 (57.47%) were on nonantidiabetic medications. Of patients receiving nonantidiabetic medications, 29 (58%) of them were on Angiotensin-converting enzyme inhibitors (ACEIs) (Table 5). Due to fear of COVID-19, 9 (10.34%) patients were unable to attend their regular follow-up and hence discontinued both antidiabetic and nonantidiabetic medications prior to the current admission.

Both antidiabetic and nonantidiabetic medications were used in the management of admitted diabetic patients. Among those, cephalosporins were the most common among anti-infectives and prescribed for 63(52.5%) patients. Similarly, antilipidemic agents 47 (39.16%) were commonly prescribed cardiovascular agents (Table 6).

### 3.7. In-Hospital Mortality

**Figure 5:**
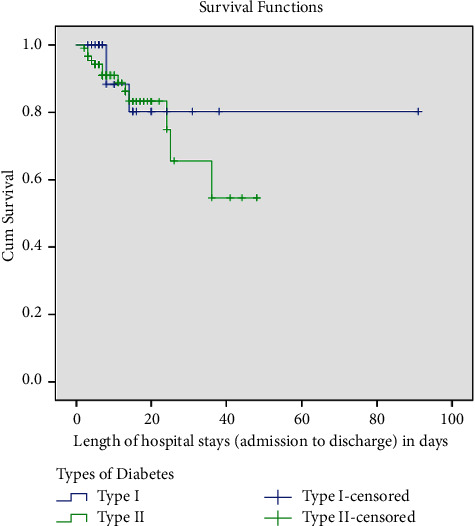
Kaplan–Meir survival curve for T1DM and T2DM patients admitted to JMC from October 01, 2020, to June 30, 2021.

In this study, 16 patients were died and the in-hospital mortality rate became 13.34%. Besides, 4 (3.33%) patients were referred to other facility and 3 (2.5%) patients were self-discharged. Of 16 in-hospital deaths, 6 (37.5%) were admitted due to infections (tuberculosis 2 (12.5%), pneumonia 2 (12.5%), meningitis, and RVI each 1 (6.25%)) and 10 (62.5%) were admitted due to various conditions, i.e.,(DKA 4 (25%), CHF 2 (12.5%), renal failure 2 (12.5%), and 1 (6.25%) each for DFU and cardiogenic shock). Five patients died within 5 days of admission while the remaining 11 died after 5 days. In this study, the mean survival time to in-hospital death was 11.5 ± 9.49 days (log-rank *p* = 0.503) (Figure 5). The median length of hospital stay was 8 (IQR 6–15.75) days.

### 3.8. Predictors of in-Hospital Mortality

Cox proportional hazard regression was conducted to identify predictors of mortality. In bivariate analysis, sex, age, residence, educational status, DM-related admission, newly diagnosed DM, DKA as admission diagnosis, history of antidiabetic medications, history of nondiabetic medications, number of comorbidities, presence of diabetic complications, and status of RBS immediate before discharge or at death were associated with death (*p* <  0.25).

However, further treatment using the multivariate Cox proportional hazard regression indicated that residence, age, presence of DKA, multiple comorbidities, and use of nonantidiabetic medication at baseline were independently associated with mortality. Accordingly, the hazard of mortality was 3.46 times higher for rural residents (AHR: 3.46; 95% CI (1.12, 9.81); *p* = 0.019)). Similarly, the hazard of mortality was increased by 3% as age increased by one unit (AHR: 1.03; 95% CI (1.001, 1.059); *p* = 0.04)). Moreover, the presence of DKA at admission increased the hazard of mortality by 5.01 times (AHR: 5.01; 95% CI (1.12, 21.88); *p* = 0.038)). Besides, the hazard of mortality was 9.65 and 14.02 times higher in patients who had five and six comorbidities (AHR: 9.65; 95% CI (1.07, 19.59); *p* = 0.043 and (AHR: 14.02; 95% CI (1.74, 21.05); *p* = 0.015)), respectively. In contrast, the risk of mortality was lowered by 86.5% among patients exposed to nonantidiabetic medications such as statins, aspirin (ASA), Angiotensin-converting enzyme inhibitors (ACEIs), Beta blockers (BBs), and calcium chanel blockers (CCBs) before admission (AHR: 0.135; 95% CI (0.04, 0.46); *p* = 0.021)) (Table 7).

## 4. Discussion

This 9-months-old longitudinal study had assessed in-hospital mortality and its predictors among diabetic patients admitted to JMC. The overall in-hospital mortality was 16 (13.34%). The type of diabetes (type 1 and type 2 diabetes) did not seem to impact the median survival time to death (*p* = 0.503). Rural residence (AHR: 3.46; 95% CI (1.12, 9.81)), age (AHR: 1.03; 95% CI (1.001, 1.059)), presence of DKA at admission (AHR: 5.01; 95% CI (1.12, 21.88)), and admission with five and six comorbidities (AHR: 9.65; 95% CI (1.07, 19.59) and (AHR: 14.02; 95% CI (1.74, 21.05), respectively, were independently associated with in-hospital mortality. In contrast, exposure to nonantidiabetic medications (AHR: 0.135; 95% CI (0.04, 0.46)) had decreased the hazard of mortality roughly by 86%.

Of 120 admitted patients, 13.34% were died. This in-hospital mortality is comparable with the report from the USA (16%) [15], Nigeria (11%) [16], and Addis Ababa, Ethiopia (10.6%) [17]. However, higher (32.5%) overall mortality was reported in another study from Nigeria (43). The discrepancy might be due to the differences in the study design and reason for admission. Among the deceased patients of our study, 37.5% were admitted due to infection. This implies that infectious conditions could be the leading causes of death. However, a study from Nigeria reported the highest mortality among patients presented with hypoglycemia, stroke, and DFU [10]. The proportion of mortality was also higher than the study from Harari region of Ethiopia (4.4%) [18] and WHO's (5.2%) report [19]. Moreover, among the deceased patients, 81.25% were diagnosed with T2DM and 56.25% of them were above the age of 60. In this study, majority of the patients were known diabetics, and consequently, they were presented with multiple comorbidities and DM-related complications. This might contribute to the amplified mortality.

The current study also identified the predictors of in-hospital mortality among admitted DM patients. Accordingly, the hazard of mortality was 3.46 times higher for rural residents (AHR: 3.46; 95% CI (1.12, 9.81)). This finding was consistent with the studies from China [20] and USA [21, 22]. In the studies from China and USA, limited access to primary health care, health literacy, lifestyle choice, and economic burden were reasons for in-hospital mortality among rural residents. In the current study, complications at admission, lower educational status, and delayed arrival to the hospital were common among rural residents, and this may attribute to the increased in-hospital mortality.

Moreover, a unit increase in age of the patient increased the hazard of the in-hospital mortality by 1.03 times (AHR: 1.03; 95% CI (1.001, 1.059)). This finding was consistent with the studies from USA [15, 23] and Jordan [24]. This could be due to the fact that old age could be more prone to increased hostile cardiovascular risk factors such as macrovascular complications, other impacts of aging, and patients' tendency to be immunosuppressed. Similarly, the risk of mortality was 5.01 times higher in patients admitted with DKA (AHR: 5.01; 95% CI: (1.12, 21.88)). The finding was in line with the studies from China [20], Nigeria [10, 25], and Addis Ababa, Ethiopia [7]. However, the finding was inconsistent with the study from Portugal where high rates of hospital mortality have been associated with DFU's [26]. The increased hazard of mortality due to DKA may be related to cerebral edema, burden of comorbidities such as precipitating factors, and some patients were also presented with septic shock. In this study, in-hospital mortality was also significantly associated with number of comorbidities. The hazard of mortality was 9.65 and 14.02 times higher in patients with five and six comorbidities (AHR: 9.65; 95% CI (1.07, 19.59)) and (AHR: 14.02; 95% CI: (1.74, 21.15)), respectively. This finding was similar to studies from Italy [27, 28], Israel [29], to Brazil [30]. Co-morbidities may be associated with increased disease severity, complicates the clinical course of diseases, and attenuates the body's natural defense mechanism against diseases by affecting multiple-body systems.

Contrarily, mortality was 86.5% lower among those exposed to nonantidiabetic medications such as statins, ASA, ACEI, BB, and CCB prior to the current admission (AHR: 0.135; 95% CI (0.04, 0.457)). This result supplemented the study from Iceland where statin use was associated with 53% reduction in all-cause mortality and 50% reduction in cardiovascular mortality in DM patients [31]. Similar finding was reported from America where statin therapy in older people (≥65 years) without CVD decreased the risk of all-cause mortality by 14%, CVD death by 20%, and stroke by 15% [32]. Moreover, the finding of this study supplemented the study conducted in America, among COVID-19 patients with DM receiving statins in whom 12% reduction in the adjusted risk of in-hospital mortality was reported [33]. It also supported the ADA guidelines recommendation which promotes the use of low-dose aspirin for diabetic patients with 10-year CVD risk ≥10% [34]. Similarly, other studies also found that ACEIs reduced all-cause mortality, cardiovascular mortality, and cardiovascular events in patients with DM [35, 36].

Though its prospective nature and longer study period (over nine months) provided better data quality, the study suffers from several limitations. First, it was a single-center study. Second, RBS was used in the study; rather than HgA1C which better describes the status of glucose control in the last three months. Third, it might be difficult to generalize the findings of this study to the entire DM population due to small sample size.

## 5. Conclusions

In this study, the rate of in-hospital mortality was high. More than one-eighth of admitted DM patients died in hospital. The study showed that rural residence, age, DKA, and having comorbidities (five and six) were the statistically significant predictors of in-hospital mortality. In contrast, the use of nonantidiabetic medications such as statins, ASA, and other antihypertensive agents before admission remained protective.

## Figures and Tables

**Table 1:** Sociodemographic characteristics of DM patients admitted to JMC from October 01, 2020, to June 30, 2021.

Variables	Category	Frequency (*n* = 120)	(%)
Sex	Male	81	67.5
	Female	39	32.5

Age in years (mean + SD)	50.21 + 19.35	120	

Residence	Rural	73	60.8
	Urban	47	39.2

Marital status	Married	92	76.7
	Single	27	22.5
	Widowed	1	0.8

Occupation	Government employee	19	15.8
	Merchant	32	26.7
	Farmer	45	37.5
	House wife	24	20

Educational status	No formal education	58	48.3
	Primary	30	25
	Secondary	11	9.2
	College and above	21	17.5

**Table 2:** Reasons for hospitalization among patients with DM admitted to JMC from October 01, 2020, to June 30, 2021.

Reason for hospitalization		Frequency (*n* = 120)	(%)
Metabolic	Diabetic ketoacidosis (DKA)	59	49.2

diseases^*∗*^ (*n* = 66)	Hyperosmolar hyperglycemic state (HHS)	5	4.17
	Hypoglycemia	2	1.67

Infections (*n* = 34)	Pneumonia	15	12.5
	Bacterial meningitis	7	5.81
	Skin and soft tissue infections (SSTI^╪^) including diabetic foot ulcer (DFU)	6	5
	Tuberculosis (TB)	4	3.33
	Retroviral infections (RVI)	2	1.66

Diseases of circulatory system (*n* = 16)	Heart failure (HF)	5	4.17
	Hypertension	4	3.33
	Stroke	4	3.33
	Acute coronary syndrome	3	2.5

Others^¥^ (*n* = 4)		4	3.33

^*∗*^DKA, HHS, and hypoglycemia. ^╪^Cellulitis, necrotizing fasciitis, septic arthritis, and osteoarthritis. ^¥^Renal failure (RF), liver disease, gastrointestinal diseases, and anemia.

**Table 3:** Prevalence of comorbidities among patients with DM admitted to JMC from October 01, 2020, to June 30, 2021.

Variables		Frequency (*n* = 120)	(%)
Presence of comorbidities	Yes	87	72.5
	No	33	27.5

*Type of comorbidities* (*n* = 87)			
Cardiovascular diseases	Hypertension	51	58.62
	Congestive heart failure (CHF)	19	21.84
	Ischemic cardiomyopathy (ICMP)	10	11.49
	Acute coronary syndrome (ACS)	10	11.49
	Stroke	8	9.19
	Hypertensive heart disease (HHD)	8	9.19
	Deep vein thrombosis (DVT)	6	6.89
	Systolic dysfunction	5	5.75
	Others^*∗*^	4	4.59

Infections	Pneumonia	35	40.23
	Urinary tract infection (UTI)	10	11.49
	Bacterial meningitis	7	8.04
	Pyelonephritis	6	6.89
	Tuberculosis (TB)	6	6.89
	COVID-19	5	5.75
	Skin and soft tissue infections (SSTI)	5	5.75
	Others^‡^	2	2.29

Kidney diseases^†^		20	22.98
Gastrointestinal diseases^╫^		10	11.49
Anemia		7	8.04
Other comorbidities^$^		2	2.29

^*∗*^Atrial fibrillation, all types of shocks, and ischemic heart disease (IHD). ^‡^Human immunodeficiency virus/acquired immunodeficiency syndrome, sepsis, and malaria. ^†^Acute kidney injury, nephrolithiasis, and chronic kidney disease. ^╫^Chronic liver disease, peptic ulcer disease; ^$^cancers (cervical and acute myeloid leukemia, hyperkalemia, and hyponatremia.

**Table 4:** Prevalence of long-term diabetic complications among patients with DM admitted to JMC from October 01, 2020, to June 30, 2021.

Variables	Frequency *n* = 120	(%)
*Presence of long-term diabetes complications*		
Yes	67	55.80
No	53	44.20

*Specific complications* (*n* *=* 67)		
Neuropathy	42	62.68
Nephropathy	30	44.77
Retinopathy	14	20.89
Diabetic foot ulcer	7	10.45

**Table 5:** Past medication history among patients with DM admitted to JMC from October 01, 2020, to June 30, 2021.

Type of medications	Frequency (*n* = 87)	(%)
*Antidiabetic medications*		
Injectable (NPH)	34	39.08
Oral glucose-lowering agents	50	57.47
Injectable + oral glucose-lowering agents	3	3.45

*Nonantidiabetic medications*	(*n* = 50)	

*Cardiovascular agents*		
Angiotensin-converting enzyme inhibitors (ACEIs)	29	58
Antilipidemic agents	21	42
Diuretics	19	38
Beta-blockers (BB)	2	4

*Drugs affecting the blood*		
Antiplatelets	12	24

**Table 6:** Patterns of medication use among diabetic patients admitted to JMC from October 01, 2020, to June 30, 2021.

Types of medications^*∗*^	Frequency *n* = 120	(%)
*Anti-infectives*		
Cephalosporins	63	52.50
Vancomycin	23	19.16
Metronidazole	19	15.83
Penicillin's	15	12.50
Tetracyclines	13	10.83
Macrolides	11	9.16
Fluoroquinolones	10	8.34
Antituberculosis drugs	8	6.67
Antifungal agents	4	3.34
Antivirals	2	1.67
Other^†^	4	3.34

*Cardiovascular agents*		
Antilipidemic agents	47	39.16
(Angiotensin-converting enzyme inhibitors (ACEIs)	40	33.34
Diuretics	26	21.67
Calcium channel blockers (CCBs)	17	14.16
Beta blockers (BBs)	16	13.34

*Drugs affecting the blood*		
Antiplatelets	41	34.16
Anticoagulants	21	17.50
Antianemics	9	7.50

*Gastrointestinal medicines*		
Antiulcer agents	16	13.34
Cathartics and laxatives	8	6.67
Antiemetics	6	5.00
Vitamins	8	6.67
Analgesics	8	6.67
Antidepressants	6	5.00
Others^$^	4	3.34

^*∗*^Drug grouped based on Ethiopian essential medicine lists, 2020. ^†^Clindamycin, meropenem. ^$^Adrenaline, dexamethasone, dopamine, and prednisolone.

**Table 7:** Bivariate and multivariable Cox proportional hazard regression to identify predictors of in-hospital mortality among DM patients admitted to JMC from October 01, 2020, to June 30, 2021.

Variables	Category	Died		CHR (95% CI)	*p* value	AHR (95% CI)	*p* value
		Yes	No				
Sex	Male	14	67	1	0.122	1	0.134
	Female	2	37	0.31 (0.07−1.37)		0.134 (0.07−1.43)	

Residence	Urban	10	36	1	0.017	1	0.019
	Rural	6	68	3.51 (1.25−9.85)		3.46 (1.12−9.81)	

Age (mean + SD)	50.21 + 19.35			1.04 (1.004−1.062)	0.027	1.03 (1.001−1.059)	0.04

Educational status	No formal education	6	52	0.198 (0.059−0.664)	0.009	0.27 (0.07−1.09)	0.06
	Primary	3	27	0.224 (0.054−0.935)	0.04	0.31 (0.06−1.57)	0.16
	Secondary	1	10	0.419 (0.05−3.503)	0.422	0.43 (0.05−3.78)	0.45
	College and above	6	15	1		1	

Type of DM	T1DM	3	29	1	0.508		
	T2DM	13	75	1.53 (0.435−5.37)			

Previous admission to JMC	Yes	8	35	0.707 (0.264−1.89)	0.49		
	No	8	69	1			

Newly diagnosed DM	Yes	2	31	2.811 (0.638−12.388)	0.172	1.85 (0.40−8.50)	0.42
	No	14	73	1		1	

DKA	Yes	2	57	4.94 (1.11−21.87)	0.036	5.01 (1.12−21.88)	0.038
	No	14	47	1		1	

Pneumonia	Yes	5	30	1.26 (0.43−3.71)	0.667		
	No	11	74	1			

Antidiabetic medication before admission	Yes	14	73	1	0.182	1	0.76
	No	2	31	0.364 (0.08−1.60)		0.78 (0.17−3.67)	

Nonantidiabetic medication use before admission (CVD drugs)	Yes	12	38	1	0.01	1	0.021
	No	4	66	0.224 (0.072−0.702)		0.135 (0.04−0.457)	

Presence of diabetic complications	Yes	16	51	0.022 (0.0003−1.445)	0.074	0.71 (0.47−5.39)	0.91
	No	0	53	1		1	

RBS immediately before discharge or death	Controlled	7	74	1	0.03	1	0.085
	Poorly controlled	9	30	3.005 (1.12−8.10)		2.42 (0.884−14.89)	

Comorbidity number	1 comorbidity	0	10	1		1	
	2 comorbidities	0	24	0.07 (0−4.07)	0.972	0.004 (0.07−9.52)	0.969
	3 comorbidities	3	17	0.297 (0.234−28.21)	0.361	2.21 (0.198−24.77)	0.519
	4 comorbidities	5	14	4.23 (1.05–38.62)	0.201	5.845 (0.495−68.99)	0.161
	5 comorbidities	3	6	10.48 (1.08−101.12)	0.04	9.65 (1.07−19.59)	0.043
	6 comorbidities	4	1	19.17 (2.11−173.81)	0.009	14.02 (1.74−21.15)	0.015

DM related admission	Yes	5	65	2.452 (0.848−7.09)	0.098	1.324 (0.402−4.365)	0.644
	No	11	39	1		1	

## Data Availability

All relevant data that support the findings of this study are within the manuscript.

## Abbreviations

DKA: Diabetic ketoacidosis
DM: Diabetes mellitus
DFU: Diabetic foot ulcer
TB: Tuberculosis
HHS: Hyperosmolar hyperglycemic state
HF: Heart failure
CVD: Cardiovascular disease
ACEIs: Angiotensin enzyme conversing inhibitors
